# Supplementary material for: Prevalence of polypharmacy and drug interactions in geriatric patients: A cross-sectional study from India
Source: PLoS One. 2026 Feb 11;21(2):e0341183. doi: 10.1371/journal.pone.0341183 (PMC12893539; doi:10.1371/journal.pone.0341183)
Supplement: S2 File — (DOCX) [file pone.0341183.s002.docx]

|  | Mean | SD |
| --- | --- | --- |
| Age | 70.39384 | 5.725001 |
| No of Drugs prescribed at treatment | 11.86301 | 4.156112 |
| No of Drugs prescribed at discharged | 7.106164 | 4.554258 |
| Drug interactions | 4.232877 | 2.506462 |
| Mild | 0.287671 | 0.984128 |
| Moderate | 3.236301 | 2.449977 |
| Severe | 0.719178 | 1.170914 |
