# Supplementary material for: Prevalence of polypharmacy and drug interactions in geriatric patients: A cross-sectional study from India
Source: PLoS One. 2026 Feb 11;21(2):e0341183. doi: 10.1371/journal.pone.0341183 (PMC12893539; doi:10.1371/journal.pone.0341183)
Supplement: Attachment — Submitted filename: Justification to reviewers comments- Polypharmacy.docx [file pone.0341183.s005.docx]

**To,**

**The Editor**

We sincerely thank you and reviewers for their valuable time, insightful comments, and constructive suggestions that have greatly improved the quality and clarity of this manuscript. We deeply appreciate their efforts in providing detailed feedback, which helped us refine our methodology, enhance the discussion, and strengthen the overall scientific rigor of our work.

**Reviewer #1:** The authors need to enhance the English used. They need to write better discussion, and they didn't mention how many they screened, they just mentioned how many they included.

**Author’s Response:** We sincerely thank the reviewer for the valuable feedback. The Discussion section has been thoroughly revised to enhance clarity, coherence, and academic tone. The English language has been improved for better readability and fluency. Additionally, we have included the number of participants screened and those finally included in the study within the methodology and results sections for greater transparency.

**Reviewer #2:** Dear authors, thank you for the work, but I need clarifications regarding my concerns.

**Comment 1:** First, I read that this paper funded by a Saudi university where the work in India, so that normal? I’m not expert in research ethics or in funding but I need clarification if that ok.

**Author’s Response:** We sincerely thank the reviewer for this observation. The study was conducted in India at Yenepoya Medical College and Hospital, where the research protocol received institutional ethical approval. The funding support from the Saudi university was provided solely to facilitate data analysis and manuscript preparation as part of an academic collaboration. No part of the funding influenced the conduct, data collection, or ethical oversight of the study. Such cross-institutional collaborations are permissible under both institutions’ ethical and research funding guidelines.

**Comment 2:** Second, you mentioned there’s restrictions on the data, I think this will affect the transparency of this research, and I don’t know if that ok with the journal and editor.

**Author’s Response:** We appreciate the reviewer’s concern regarding data transparency. The data used in this study are subject to institutional confidentiality policies of Yenepoya Medical College and Hospital, which restrict public sharing of patient-level information to protect privacy. However, aggregated or anonymized data can be made available upon reasonable request to the corresponding author, following approval from the institutional ethics committee. This approach ensures both ethical compliance and research transparency in accordance with journal standards.

**Comment 3:** I think the abstract needs to be rewritten, it doesn’t have enough of essential information, like short background, name of the institution, number of participants (in numbers) so I advise to rewrites with more details.

**Author’s Response:** We thank the reviewer for this valuable suggestion. The abstract has been revised to include additional essential details, such as a concise background, the name of the study institution, and the total number of participants (n=310). The revised version provides a clearer summary of the study design, methodology, and key findings, thereby improving completeness and clarity in line with the journal’s requirements.

**Comment 4:** The methodology section is confusing. Please rewrite in more arrangement and make the main sections.

**Author’s Response:** The methodology has been comprehensively reorganized under clear subheadings — Study Design and Setting, Study Population and Definitions, Sample Size and Sampling Technique, Inclusion and Exclusion Criteria, Data Collection Procedure, and Statistical Analysis — to improve clarity and logical flow.

**Comment 5:** You need to justify the selection of these settings and more information about it.

**Author’s Response:** Yenepoya Medical College and Hospital was chosen because it caters to a heterogeneous population from urban, suburban, and rural regions, allowing for a diverse and representative sample of elderly patients. Its comprehensive inpatient and outpatient facilities made it ideal for assessing patterns of polypharmacy in varied clinical contexts.

**Comment 6:** Also justify the sampling at all. The calculator, size, and technique.

**Author’s Response:** The sample size (n=310) was calculated using the Raosoft online calculator with standard assumptions (95% CI, 5% margin of error, 50% response rate) to ensure representativeness. A simple random sampling technique was adopted to minimize selection bias and ensure equitable inclusion of eligible inpatients and outpatients.

**Comment 7:** I didn’t get the data collection process. You interviewed the patients? Or get the data from the health records? As I said before please rewrite the methods in clear and arranged style.

**Author’s Response:** Data were obtained exclusively from hospital electronic medical records (EHRs) and verified through patient files, not interviews.

**Comment 8**: In the limitation section, Please separates the limitation and recommendations.

**Author’s Response:** We appreciate the reviewer’s suggestion. The section has been revised to clearly separate the limitations and recommendations sections in the updated manuscript.
